# Supervised and Unsupervised Screen Time and Its Association With Physical, Mental, and Social Health of School-Going Children in Dhaka, Bangladesh: Cross-Sectional Study

**DOI:** 10.2196/62943

**Published:** 2025-01-14

**Authors:** Shahria Hafiz Kakon, Tanjir Rashid Soron, Mohammad Sharif Hossain, Rashidul Haque, Fahmida Tofail

**Affiliations:** 1 Infectious Diseases Division, International Centre for Diarrheal Disease Research, Bangladesh, 68, Shaheed Tajuddin Ahmed Sarani, Mohakhali, Dhaka, 1212, Bangladesh, 880 1726428760; 2 Telepsychiatry Research and Innovation Network Ltd, Dhaka, Bangladesh; 3 Maternal and Child Nutrition, Nutrition Research Division, International Centre for Diarrheal Disease Research, Bangladesh, Dhaka, Bangladesh

**Keywords:** screen time, parental supervision, Strength and Difficulties Questionnaire, Spencer Children Anxiety Scale, Pittsburgh Sleep Quality Scale, children, sleep quality, headache, behavioral problems

## Abstract

**Background:**

Children’s screen time has substantially increased worldwide, including in Bangladesh, especially since the pandemic, which is raising concern about its potential adverse effects on their physical, mental, and social health. Parental supervision may play a crucial role in mitigating these negative impacts. However, there is a lack of empirical evidence assessing the relationship between parental screen time supervision and health outcomes among school children in Dhaka, Bangladesh.

**Objective:**

We aimed to explore the association between supervised and unsupervised screen time on the physical, mental, and social health of school-going children in Dhaka, Bangladesh.

**Methods:**

We conducted a cross-sectional descriptive study between July 2022 and June 2024. A total of 420 children, aged 6‐14 years, were enrolled via the stratified random sampling method across three English medium and three Bangla medium schools in Dhaka. Data were collected through a semistructured questionnaire; anthropometry measurements; and the Bangla-validated Strength and Difficulties Questionnaire (SDQ), Pittsburgh Sleep Quality Index (PSQI) Scale, and Spencer Children Anxiety Scale (SCAS).

**Results:**

A total of 234 out of 420 students (56%) used digital screen devices without parental supervision. We did not find a substantial difference in the duration of the daily mean use of digital devices among the supervised students (4.5 hours, SD 2.2 hours) and the unsupervised students (4.6 hours, SD 2.4 hours). According to the type of school, English medium school children had a mean higher screen time (5.46 hours, SD 2.32 hours) compared to Bangla medium school children (3.67 hours, SD 2.00 hours). Headache was significantly higher among the unsupervised digital screen users compared to those who used digital screens with parental supervision (175/336 students, 52.1% versus 161/336 students, 47.9%; *P*<.003). Moreover, students who used digital screens without parental supervision had poor quality of sleep. Behavioral problems such as conduct issues (119/420 students, 28.3%) and peer difficulties (121/420 students, 28.8%) were observed among the participants. However, when comparing supervised and unsupervised students, we found no statistically significant differences in the prevalence of these issues.

**Conclusions:**

The findings of the study showed that the lack of screen time supervision is associated with negative health effects in children. The roles of various stakeholders, including schools, parents, policy makers, and students themselves, are crucial in developing effective guidelines for managing screen use among students. Further research is needed to demonstrate causal mechanisms; identify the best interventions; and determine the role of mediators and moderators in households, surroundings, and schools.

## Introduction

To what extent can parental supervision mitigate the effects of excessive digital screens in school-going adolescents? This is becoming an increasingly common concern of parents and researchers, as digital screens fill our living spaces and take up our quality time. A recent study showed that daily screen time for users aged 16‐64 years has increased to 6 hours 37 minutes worldwide [1]. Across age groups, 44% of all waking hours are spent on screens [1]. In 2020, the World Health Organization released a milestone report with detailed guidelines on sedentary behavior including screen time for children under the age of 5 years, defining screen time as time spent passively watching screen-based entertainment (TV, computer, or mobile devices) [2]. The American Academy of Child and Adolescent Psychiatry agreed with the findings, recommending less than 2 hours of daily screen time for children aged under 5 years, but for children aged 6 years and older, recommending “healthy habits” and to “limit activities that include screens” [3]. At present, no concrete guidelines have been presented for adolescents, although health concerns are just as pressing for them [4].

Higher sedentary behavior such as screen time is associated with a range of physical and mental health issues, including headaches, myopia, obesity, sleep disorders, behavioral disorders, and anxiety [4-8]. A study of 5844 children around the world found that children averaged 8.6 hours of daily sedentary time, associated with poor weight status and physical inactivity [9]. Lauricella and Cingel [10] found one of the most reliable predictors of higher screen time among adolescents to be parental media use, along with weaker associations with parental attitudes to technology and screen time rules.

In Asia, higher incomes and a rising middle class have led to longer exposure to screen time (>2 h a day) for children and adolescents [11,12]. Without parental intervention, excessive screen time exposure has been associated with less sleep duration among Asian preschoolers, but Lin et al [13] found parental education and awareness, among best practices, to be an effective intervention against these effects.

Bangladesh’s rising socioeconomic status has contributed to an explosion in digital device access and usage across demographics [14]. Khan and Burton [15] found that almost 80% of Bangladeshi adolescents have high recreational screen time, reported at 4.0 (SD 2.2) hours per day on average, with the mean values of 4.3 (SD 2.4) hours for boys and 3.6 (SD 2.3) hours for girls. High screen time was associated with sleep disturbance and higher family income, among other factors [15]. Anjum et al [16] found a higher incidence of depressive symptoms in adolescents with higher screen time exposure (>2 h per day), alongside other physical and mental health concerns including sleep disturbance and mood disorders prior to the COVID-19 pandemic.

During the COVID-19 lockdown, Bangladeshi children began to spend much more time on the internet, for entertainment, communication, and education [17-19]. Koly and colleagues [20] noted worsening psycho-social health of school-going students in this period, associated with quarantine adaptations and difficulties with online learning. Simultaneously, Rashid and colleagues [21] found physical and mental health deterioration among secondary school students, with symptoms including headaches, backaches, visual and sleep disturbance, and depression. During the pandemic, online learning became a necessity, leading to a significant increase in children’s digital screen use. While it ensured educational continuity, this shift also contributed to extended screen time, potentially indicating the physical, mental, and emotional health risks associated with excessive screen exposure [4,22].

Following the pandemic, increased digital screen time did not lessen, leading to significant adverse consequences on adolescent health [23,24]. For instance, Shuvo and Biswas [25] note that when electronic device exposure overlaps with eating times and habits, there is an increased likelihood of obesity. Meanwhile, heavier reliance on technology for education, entertainment, and socialization after COVID is associated with increased anxiety, difficulty sleeping, addiction, and various sociobehavioral difficulties [23,24,26].

In Bangladesh, there is a dearth of comprehensive academic research on the effects of screen time on adolescents, despite the fact that there is a substantial amount of empirical evidence around the world [4,22,27-34]. Furthermore, as the results are utilized to create thorough screen-use guidelines, these studies have significant policy ramifications. Furthermore, while parental supervision is crucial in preventing excessive screen time, researchers found that inappropriate methods to discipline children and adolescents may increase long-term screen use and exaggerate other adverse psychosocial effects [24,35]. Therefore, this study aimed to distinguish between the effects of supervised and unsupervised screen time on the physical, mental, and social health of Bangladeshi schoolchildren.

## Methods

### Study Setting, Participants, and Sampling

This was a cross-sectional descriptive study and adopted a quantitative approach. The survey was conducted between July 2022 to June 2024. We purposively selected 6 schools from the list of all schools in Dhaka North and South city corporations to ensure the equal distribution of the Bangla and English medium schools. Moreover, we considered the socioeconomic, geographic, and feasibility of data collection. During the school selection, with the approval of the school authorities, we considered children aged between 6 to 14 years who were in class 2 to class 8 and who had more than 2 hours of screen time each day as the qualifying criteria for sample inclusion. Smartphones, tablets, laptops, personal computers, gaming consoles, televisions, and portable gaming devices were among the acceptable gadgets. Children with preexisting conditions, such as physical or psychological impairment, were excluded from the study, as this might have influenced the results. We used a stratified random sampling method to recruit a total of 420 students, by including 70 students from each school (ie, 10 students from each grade from grades 2 to 8).

### Data Collection Procedure

We collected data through face-to-face interviews with parents and students on school grounds. The day prior to the interview, study staff scheduled the appointment time with parents, and the class teacher brought the students during the lunch break. A half-hour interview period was allotted for students and an hour for parents. To check for differences in accuracy between staff and trainers (ie, psychologists who trained all the staff on each of the psychometric tools used in this study), 30 pilot tests were performed prior to the start of data collection. Interrater reliability was measured using the Cohen kappa coefficient to quantify the level of agreement between each of the three staff and trainers (n=30 nonstudy participants/staff) for all the Bangla-validated scales. On average, the calculated Cohen kappa values ranged between 0.84 and 0.89 (scores for 30 nonstudy participants/staff-trainer pairs), indicating strong agreement among the staff.

All data collection forms were checked at the field site for completeness, accuracy, and consistency. The quality control team was responsible for regular observations at school, and identical forms and tools were used across the duration of the study. Investigators personally traveled to the sites weekly to ensure proper field implementation.

### Study Instruments

#### Screen Time

A semistructured questionnaire was developed to collect information about screen time from both parents and students. The parents’ questionnaires included socioeconomic factors, pattern and quantity of child screen time usage, gadget-using behavior of the children, and parents’ mediating role in screen time exposure (supervised vs unsupervised). The student’s questionnaire included the type of device use, time and pattern of use, and purpose of device use. Screen time was considered to be any time spent engaging with content in front of a digital device with an electronic screen, including but not limited to iPads, gaming consoles, laptops, smartphones, tablets, and desktop devices. Students were categorized into supervised versus unsupervised based on the self-reporting information regarding the supervision. In this study, supervised screen time is defined as when parents are aware of their child’s digital device usage, including what they are doing, how much time they spend, and the type of content they consume. In contrast, unsupervised screen time occurs when parents lack awareness of their child’s activities, duration of use, and the content they engage with on digital devices. Similar definitions have been used in other studies [36,37].

#### Physical Health

Anthropometric measurements (weight and height) were collected from students on school premises during data collection. Measurements were calibrated daily before data collection to ensure standardization. The BMI (kg/m^2^) was calculated and converted to z-scores, then categorized according to cut-off points given by the World Health Organization BMI-for-age growth chart for ages 5 to 19 years: underweight (<15 kg/m^2^, −1 SD), normal weight (15‐25 kg/m^2^), overweight (25‐30 kg/m^2^, +1 SD), obese (30‐40 kg/m^2^, +2 SD), and morbidly obese (>40 kg/m^2^, +4 SD) [38]. Additional physical health issues, such as blurred vision, headaches, indigestion, backache, and neck pain, were also asked of the students via a questionnaire. The students’ physical health responses were initially categorized into four groups: “No,” “Sometimes,” “Often,” and “Most of the time.” These categories were then consolidated into two broader groups: “No” and “Yes,” with “Yes” encompassing the responses “Sometimes,” “Often,” and “Most of the time.”

#### Mental and Social Health

Age-appropriate Bangla-validated versions of the following scales were used to find well-being indicators for children’s mental and social health.

##### Strength and Difficulties Questionnaire

This is a behavioral questionnaire (25 questions) designed to identify a combination of positive and negative attributes across 5 dimensions–emotional symptoms, conduct problems, hyperactivity or attention deficit, peer relationship problems, and prosocial behavior. The sum of the scores of the four negative behavior subscales represents the children’s general difficult behavior with a maximum score of 40, whereas the maximum score for prosocial behavior is 10. Gustin and colleagues have verified the Strength and Difficulties Questionnaire (SDQ) against independent clinical diagnoses of Bangladeshi children [39,40].

##### Spencer Children Anxiety Scale

This scale is used to evaluate symptoms relating to separation anxiety, social phobia, obsessive-compulsive disorder, panic, agoraphobia, generalized anxiety, and fear of injury. Goodman et al have validated the Spencer Children Anxiety Scale (SCAS) as a reliable instrument for Bangla-speaking communities [41].

##### Pittsburgh Sleep Quality Index

This questionnaire has components spanning several subcategories such as subjective sleep quality, latency, duration, habitual efficiency, disturbances, sleeping medication, and daytime dysfunction. Mamun et al [42] have successfully used the Pittsburgh Sleep Quality Index (PSQI) to identify sleep-related concerns among Bangladeshi students.

### Statistical Analysis

Data were entered via IBM SPSS version 20.0 (IBM Corp) and analyzed on Stata version 15.1 (StataCorp LLC). Categorical data were represented as frequency numbers and percentages, while continuous data with reasonably normal distributions were summarized as means and SDs. Nonnormal continuous data were instead summarized as medians and IQRs. Participants were categorized into two main subgroups, supervised and unsupervised. Subsequently, a comparative analysis was performed across various factors, including demographic characteristics, amount of screen time, and health metrics. To assess differences across groups, the chi-squared test for independence was employed for categorical data, and the 2-tailed unpaired *t* test for differences between proportions was used for continuous data with nearly normal distributions. A significance threshold of *P*<.05 was used to assess statistical significance.

### Ethical Considerations

This study was approved by the Institutional Review Board of icddr,b (protocol number: PR-22002). Written informed consent was obtained from all parents, and confidentiality and anonymity were maintained throughout the study. Children aged above 11 years provided assent in addition to their parents’ consent. All the respondents were informed in Bengali about their rights related to their voluntary participation in the study as well as their right to withdraw from the interview at any time during the interview.

## Results

A total of 420 students were enrolled based on the screening criteria. Of them, 186 (44%) children were supervised and 234 (56%) children were unsupervised. Table 1 represents the demographic summary statistics of the population. The students were between 6 and 14 years, with a mean age of 10.9 (SD 1.9) years; 207 out of the 420 children (49.3%) were girls. Of the 420 children, 292 (69.5%) belonged to single-family households. In families with 1‐2 children, 161 out of 288 children (55.9%) were unsupervised, while for families with more than 3 children, 73 out of 132 children (55.3%) were unsupervised.

**Table 1.** Demographic and socioeconomic information of study participants.

Characteristics	Overall (N=420)	Supervised (n=186)	Unsupervised (n=234)
Child’s age (years)			
Mean (SD)	10.9 (1.9)	11.0 (1.9)	10.8 (1.9)
Child’s sex, n (%)			
Male	213 (50.7)	101 (47.4)	112 (52.6)
Female	207 (49.3)	85 (41.1)	122 (58.9)
Family type, n (%)			
Single	292 (69.5)	132 (45.2)	160 (54.8)
Joint	128 (30.5)	54 (42.2)	74 (57.8)
Number of children in the family, n (%)			
1‐2	288 (68.6)	127 (44.1)	161 (55.9)
≥3	132 (31.4)	59 (44.7)	73 (55.3)
Average monthly income, n (%)			
<BDT^a^ 50,000 (<US$ 420)	137 (32.6)	57 (41.6)	80 (58.4)
BDT 50,000‐100,000 (>US$ 420 to <US$ 840)	144 (34.3)	63 (43.8)	81 (56.2)
>BDT 100,000 (>US$ 840)	139 (33.1)	66 (47.5)	73 (52.5)

a BDT: Bangladeshi taka.

The mean total daily screen time for the entire population was 4.6 (SD 2.3) hours. The large SD relative to the mean indicates widely varying screen habits among the study population. The range of screen time for the unsupervised group was 0.3‐15 hours and that for the supervised group was 0‐12 hours. The mean total daily screen time for the unsupervised group was 4.6 (SD 2.4) hours, slightly higher than the supervised group’s mean of 4.5 (SD 2.2) hours. These large deviations imply factors other than supervision contribute more to total screen time.

Multimedia Appendix 1 shows the mean daily screen time spent by children categorized by age, sex, and type of school. No significant difference was observed in the mean daily screen time between different age groups (6‐10 years vs 11‐14 years) or between male and female participants. However, children attending English medium schools had a significantly higher average daily screen time (5.4 hours) compared to those attending Bangla medium schools (3.6 hours).

Table 2 shows the prevalence of physical symptoms among students with supervised and unsupervised use of digital devices. Overall, out of 420 students, 391 (93.1%) students experienced blurred vision, 341 (81.2%) reported abdominal pain, 336 (80%) had headaches, and 327 (77.9%) experienced dry eyes or soreness. Considering all the health issues, a higher proportion of unsupervised students experienced these problems compared to supervised students. Headaches were significantly more prevalent among the unsupervised group (175/336 children, 52.1%) than the supervised group (161/336 children, 47.9%; *P*<.003). All the data are shown in Table 2.

**Table 2.** Prevalence of physical symptoms among study participants with supervised and unsupervised use of digital screens and devices.

Physical symptoms	Frequency (N=420), n (%)	Supervised (n=186), n (%)	Unsupervised (n=234), n (%)	*P* value
Eye problems	150 (35.7)	64 (42.7)	86 (57.3)	.62
Dry eye or soreness	327 (77.9)	150 (45.9)	177 (54.1)	.22
Blurred vision	391 (93.1)	177 (45.3)	214 (54.7)	.14
Hearing difficulty	14 (3.3)	6 (42.9)	8 (57.1)	.91
Indigestion or gas	276 (65.7)	118 (42.8)	158 (57.2)	.38
Headache	336 (80.0)	161 (47.9)	175 (52.1)	.003
Neck pain	192 (45.7)	86 (44.8)	106 (55.2)	.85
Abdominal pain	341 (81.2)	150 (44.0)	191 (56.0)	.80
Back or any other musculoskeletal problem	134 (31.9)	63 (47.0)	71 (53.0)	.44
Diabetes	4 (1.0)	0 (0.0)	4 (100.0)	.07
Change in appetite	204 (48.6)	86 (42.2)	118 (57.8)	.39
Sleep issues	82 (19.5)	32 (39.0)	50 (61.0)	.29

Table 3 shows a mean BMI of 19.3 (SD 4.7) kg/m^2^ for the study population. There was a slight difference in the mean BMI between the supervised (mean 19.6 kg/m^2^, SD 4.9 kg/m^2^) and unsupervised (mean 19.1 kg/m^2^, SD 4.4 kg/m^2^) groups (*P*=.26 indicates statistical insignificance). Overall, out of 420 children, 335 (80.1%) children were healthy, 21 (5%) were underweight, and 42 (10.1%) were overweight/obese. No statistically significant differences were observed in the distribution of BMI between the supervised and unsupervised categories.

**Table 3.** Association of BMI with supervised and unsupervised digital screen use.

Characteristics	Overall (N=420)	Supervised (n=186)	Unsupervised (n=234)	*P* value
BMI, kg/m^2^				.26
Mean (SD)	19.3 (4.7)	19.6 (4.9)	19.1 (4.4)	
BMI category, n (%)				.50
Underweight	21 (5.0)	9 (42.9)	12 (57.1)	
Healthy weight	335 (80.1)	143 (42.7)	192 (57.3)	
Overweight	42 (10.1)	21 (50.0)	21 (50.0)	
Obesity	16 (3.8)	9 (56.2)	7 (43.8)	
Severe obesity	4 (1.0)	3 (75.0)	1 (25.0)	

Table 4 shows the association of anxiety with supervised and unsupervised digital screen use. Out of 420 children, 414 (98.6%) children were identified within the normal range. Just 1 of the 6 children fell into the high anxiety range; only 1 was left unsupervised and the other 5 were in the supervised group. Despite the fact that there was a significant difference (*P*<.05), the small number of individuals in this group could provide an inaccurate correlation. It is recommended to conduct additional research before drawing any conclusions. There were no discernible variations between the supervised and unsupervised proportions for any specific anxiety subcategory on the SCAS.

Multimedia Appendix 2 shows the results of the SDQ scale in the 420 students; of the 420 students, 119 students (28.3%) had conduct problems, 121 students (28.8%) had peer problems, 66 students (15.7%) reported emotional problems, 73 students (17.4%) experienced hyperactivity, and 28 students (6.7%) reported prosocial behaviors. Based on the borderline/abnormal results shown in Multimedia Appendix 2, Table 5 was prepared, and it shows the distribution of supervised and unsupervised children categorized as having borderline/abnormal results for digital screen use based on the SDQ subcategories (emotional symptoms, conduct problems, hyperactivity or attention deficit, peer relationship problems, and prosocial behavior).

The percentage of unsupervised children was greater than that of supervised children among those classified as having borderline or abnormal results. The most common abnormality was peer relationship problems among the children. Of the 420 students, 121 (28.8%) had peer relationship problems: 55 students (45.38%) were supervised and 66 students (55.6%) were unsupervised. Less borderline/abnormal results were found considering prosocial behavior. Of the 420 students, 28 (6.7%) were identified with abnormal/borderline results: 11 (39.29%) were supervised and 17 (60.71%) were unsupervised.

**Table 4.** Association of anxiety with supervised and unsupervised digital screen use by using the Spencer Children Anxiety Scale (SCAS).

SCAS	Overall (N=420), n (%)	Supervised (n=186), n (%)	Unsupervised (n=234), n (%)	*P* value
Overall				.05
Normal range	414 (98.6)	181 (43.7)	233 (56.3)	
Elevated range	6 (1.4)	5 (83.3)	1 (16.7)	

**Table 5.** Distribution of supervised and unsupervised children categorized as borderline/abnormal for digital screen use based on the Strength and Difficulties Questionnaire subcategories (emotional symptoms, conduct problems, hyperactivity, peer relationship problems, and prosocial behavior).

Category	Supervised (%)	Unsupervised (%)
Emotional symptoms	43.94	56.66
Conduct problems	45.38	54.62
Hyperactivity	47.95	52.05
Peer relationship problems	45.45	54.55
Prosocial behavior	39.29	60.71

Table 6 shows that a majority of children reported good quality of sleep on the PSQI (358/420, 85.2%). Of those who reported bad sleep, the proportions were similar between supervised and unsupervised children (14% of supervised children vs 15.4% of unsupervised children). Nonetheless, a potentially significant result was found on comparing the mean total sleeping durations: supervised children sleep on average for 7.7 (SD 1.5) hours, compared to 7.4 (SD 1.6) hours for unsupervised children (against an overall mean of 7.6, SD 1.6). However, the *P* value showed borderline significance; furthermore, if the children were subcategorized by the average hours of sleep (>7, 6‐7, 5‐6, and <5 hours), no significant difference was found across any subgroup between supervised and unsupervised children.

**Table 6.** Association of quality of sleep using the Pittsburgh Sleep Quality Index (PSQI) scale with supervised and unsupervised digital screen use.

PSQI scale	Overall (N=420)	Supervised (n=186)	Unsupervised (n=234)	*P* value
Sleep status, n (%)				.69
Good sleep (global PSQI score ≤5)	358 (85.2)	160 (44.6)	198 (55.3)	
Bad sleep (global PSQI score >5)	62 (14.8)	26 (14.0)	36 (15.4)	
Sleep time				.05
Mean bedtime, PM	11:17	11:11	11:21	
Mean wake time, AM	7:19	7:24	7:16	
Total sleeping duration (h), mean (SD)	7.6 (1.6)	7.7 (1.5)	7.4 (1.6)	
Sleep quality, n (%)				.18
Very good	202 (48.1)	82 (44.1)	120 (51.3)	
Fairly good	199 (47.4)	94 (50.5)	105 (44.9)	
Fairly bad	17 (4.0)	10 (5.4)	7 (3.0)	
Very bad	2 (0.5)	0 (0.0)	2 (0.9)	
Sleep latency, n (%)				.85
0	100 (23.8)	41 (22.0)	59 (25.2)	
1‐2	207 (49.3)	94 (50.5)	113 (48.3)	
3‐4	105 (25.0)	48 (25.8)	57 (24.4)	
5‐6	8 (1.9)	3 (1.6)	5 (2.1)	
Sleep duration, n (%)				.06
>7 hours	237 (56.4)	112 (60.2)	125 (53.4)	
6‐7 hours	123 (29.3)	53 (28.5)	70 (29.9)	
5‐6 hours	44 (10.5)	19 (10.2)	25 (10.7)	
<5 hours	16 (3.8)	2 (1.1)	14 (6.0)	
Habitual sleep efficiency, n (%)				.40
>85%	393 (93.6)	176 (94.6)	217 (92.7)	
75‐84%	25 (6.0)	10 (5.4)	15 (6.4)	
65‐74%	2 (0.5)	0 (0.0)	2 (0.9)	
<65%	0 (0.0)	0 (0.0)	0 (0.0)	
Sleep disturbances, n (%)				.80
0	38 (9.0)	18 (9.7)	20 (8.6)	
1‐9	364 (86.7)	161 (86.6)	203 (86.8)	
10‐18	17 (4.0)	7 (3.8)	10 (4.3)	
19‐27	1 (0.2)	0 (0.0)	1 (0.4)	
Use of sleeping medication, n (%)				.30
Not during the past month	417 (99.3)	186 (100.0)	231 (98.7	
Less than once a week	2 (0.5)	0 (0.0)	2 (0.9)	
Once or twice a week	1 (0.2)	0 (0.0)	1 (0.4)	
Three or more times a week	0 (0.0)	0 (0.0)	0 (0.0)	
Daytime dysfunction, n (%)				.88
0	283 (67.4)	124 (66.7)	159 (68.0)	
1‐2	121 (28.8)	54 (29.0)	67 (28.6)	
3‐4	13 (3.1)	7 (3.8)	6 (2.6)	
5‐6	3 (0.7)	1 (0.5)	2 (0.9)	

## Discussion

### Principal Results and Comparison With Prior Works

This study enrolled a total of 420 children, with 186 (44%) supervised and 234 (56%) unsupervised children. Unsupervised children had a slightly higher mean screen time of 4.6 (SD 2.4) hours compared to supervised children who had a mean screen time of 4.5 (SD 2.2) hours. Additionally, the study found a higher prevalence of physical symptoms and slightly different BMI distributions among unsupervised children. No significant differences in sleep quality were observed between supervised and unsupervised children, although supervised children slept slightly longer. A greater percentage of unsupervised children were categorized as having borderline or abnormal findings based on the SDQ subcategories (emotional symptoms, conduct problems, hyperactivity or attention deficit, peer relationship problems, and prosocial behavior).

Significant variations depending on the type of education were found in this study, which examined key facets of the role of parental supervision in digital screen use among students in Dhaka city. Although teenagers attending school spent a mean of 4.6 (SD 2.3) hours a day on screens, there was no discernible difference in the amount of time spent on screens between students who were under supervision and those who were not. Nevertheless, when stratified by the type of education, students in Bengali medium schools had screen time of 3.67 hours, while students in English medium schools showed considerably higher screen time (5.46 hours). This discrepancy could have several causes. In Bangladesh, English medium students often come from higher-income households that can afford the higher costs of English medium education, making them more likely to have access to personal digital devices. A similar indication was provided in an article published by The Daily Star [43]. Additionally, English medium students are typically Ordinary level (O level) or Advanced Level (A level) candidates, an internationally recognized qualification that is considered to be the equivalent of Cambridge IGCSE and UK General Certificate of Secondary Education; the preparation for these international-standard examinations necessitates greater screen exposure as part of their learning process [44]. Despite the differences in total screen time, the study did not find any correlations between parental education or income level and screen time supervision. Another study also showed the same findings that no significant association was observed between parental education or income level and screen time supervision [45].

Such trends are concerning given the established negative effects of excessive screen time on adolescent health, including obesity, diabetes, poor sleep, and increased risks of depression and anxiety [5-9]. Effective parental supervision is known to reduce screen time and mitigate these adverse effects. Parent-child interaction improved prosocial behavior and reduced psychosocial difficulties while contributing to healthier body mass indices and better sleep [11]. Furthermore, the findings of this study align with the results from previous studies [7, 9]. The empirical evidence found that parental mediation and active participation are associated with improved physical health outcomes, including BMI and sleep duration [7,9]. School-going participants in this study also reported a range of health concerns, with blurred vision, abdominal pain, headaches, dry or sore eyes, and indigestion being the most commonly noted. Such impacts are also reported by several previous studies [46-48]. A notable difference was observed between supervised and unsupervised groups specifically for headaches, with a higher proportion of unsupervised students reporting this issue. A previous study also reported that unsupervised children are more vulnerable to associated health issues [49]. Although supervision did not significantly impact other physical indicators, the findings suggested a potential association with blurred vision and diabetes. However, the small number of patients with diabetes limits the interpretability of these findings. Therefore, further research is needed to explore the effects of different supervision methods on these health concerns and to identify any potential correlations.

In contrast, the statistical significance indicates that factors other than supervision, such as lifestyle, might have a greater impact on other physical issues. For instance, a sizable percentage (28.3%) of kids showed conduct and peer relationship problems when they had results in the borderline or abnormal range on the SDQ. Despite these findings, no significant differences were observed between supervised and unsupervised groups across the SDQ subcategories, suggesting that these behavioral issues may be influenced by factors beyond supervision, including lifestyle and home or school conditions.

Furthermore, we identified a concern that supervision might exacerbate anxiety. This is because, among the 6 children who reported overall anxiety, only 1 was unsupervised. Although the result is statistically significant, this finding should be interpreted with caution due to the small sample size. Collectively, these observations highlight a significant deficiency in effective parental guidance and supervision methods among contemporary urban families in Dhaka. Therefore, further research is needed to explore the specific supervision strategies employed by parents and their impact, with the aim of developing guidelines for healthy technology use.

Finally, we explored a potential correlation between parental supervision of digital screen use and the mean total bedtime based on PSQI data, with the supervised group having slightly more sleep on average compared to their counterpart. However, when analyzing total sleep duration and examining differences related to supervision across various subgroups, the significance diminished, indicating that the observed differences were not conclusive. Specifically, the proportions of children who slept more than 7 hours were similar between the supervised and unsupervised groups, and these proportions did not differ meaningfully from the overall study sample. Given these borderline findings, further investigation is warranted to explore the potential associations between supervision and sleep patterns.

### Limitations

This study has several limitations. First, inconsistencies emerged between the parents’ and children’s reports, as many parents used a broad definition of “supervised,” such as occasional checks or limited awareness of the child’s activities on the device. As the children’s reports were found to be more reliable predictors, final supervision categorization was based on the children’s statements. A similar approach has been followed previously [44]. Second, the study’s scope was limited to 6 schools (3 Bangla and 3 English medium schools), which may not be representative of the broader variation between urban and rural settings or differing school resources. Third, data on technological interactions and parental mediation were collected via face-to-face interviews, which may have introduced recall bias. Finally, discrepancies between parent and child reports could not be independently verified. Future research should include diverse populations and settings to provide a more comprehensive understanding of screen time impacts.

### Strengths

Despite these limitations, the strength of this study lies in its pioneering exploration of the association of parental supervision with the physical, mental, and social well-being of students in Bangladesh. Additionally, by including students from both Bangla and English medium schools, the study provides valuable comparative insights. The findings are expected to motivate and inform parents, policy makers, and educational authorities, highlighting the need for enhanced supervision and education to promote healthier and more balanced lifestyles for students. Further research is required that may explore causal relationships through experimental or longitudinal designs.

### Conclusions

The results of this study enhance our understanding of how to mitigate the negative impacts of unsupervised screen time on students’ well-being. Effective guidelines for managing screen use require the involvement of multiple stakeholders: schools, parents, policy makers, and the students themselves. Schools can play a crucial role in educating students about safe screen use and enforcing balanced screen time through workshops and seminars. Parents need to be informed about the risks of excessive screen time and the benefits of active supervision, adopting strategies to enhance their children’s well-being. Additionally, accessible mental health services, including counseling and support groups, can help students manage stress and anxiety related to screen time. Conducting further research to develop comprehensive screen time guidelines is essential for promoting the health and well-being of future generations.

## Supplementary material

10.2196/62943 Multimedia Appendix 1 Mean daily screen time (hours) by sociodemographic characteristics.

10.2196/62943 Multimedia Appendix 2 Distribution of normal and borderline/abnormal responses for the student behavior on the Strength and Difficulties Questionnaire (SDQ) scale.

## Abbreviations

PSQI: Pittsburgh Sleep Quality Index
SCAS: Spencer Children Anxiety Scale
SDQ: Strength and Difficulties Questionnaire
