# Supplementary material for: Supervised and Unsupervised Screen Time and Its Association With Physical, Mental, and Social Health of School-Going Children in Dhaka, Bangladesh: Cross-Sectional Study
Source: JMIR Pediatr Parent. 2025 Jan 14;8:e62943. doi: 10.2196/62943 (PMC11749080; doi:10.2196/62943)
Supplement: Multimedia Appendix 1 [file pediatrics-v8-e62943-s001.docx]

| Characteristics | N =420 (%) | Mean ± SD | *P* value |
| --- | --- | --- | --- |
| Child's Age |  |  | .35 |
| 6-10 Years | 181 (43.1) | 4.44 ± 2.13 |  |
| 11-14 Years | 239 (56.9) | 4.66 ± 2.47 |  |
| Child's Sex |  |  | .48 |
| Male | 213 (50.7) | 4.65 ± 2.36 |  |
| Female | 207 (49.3) | 4.49 ± 2.30 |  |
| Type of School |  |  | <.001 |
| English Medium | 210 (50.0) | 5.46 ± 2.32 |  |
| Bangle Medium | 210 (50.0) | 3.67 ± 2.00 |  |
