# Supplementary material for: Supervised and Unsupervised Screen Time and Its Association With Physical, Mental, and Social Health of School-Going Children in Dhaka, Bangladesh: Cross-Sectional Study
Source: JMIR Pediatr Parent. 2025 Jan 14;8:e62943. doi: 10.2196/62943 (PMC11749080; doi:10.2196/62943)
Supplement: Multimedia Appendix 2 [file pediatrics-v8-e62943-s002.docx]

| **SDQ scale** | **Overall**  **N =420 (%)** |
| --- | --- |
| **Emotional Problem** |  |
| Normal | 354 (84.3) |
| Borderline/Abnormal | 66 (15.7) |
| **Conduct Problem** |  |
| Normal | 310 (71.7) |
| Borderline/Abnormal | 119 (28.3) |
| **Hyperactivity** |  |
| Normal | 347 (82.6) |
| Borderline/Abnormal | 73 (17.4) |
| **Peer Problem** |  |
| Normal | 299 (71.2) |
| Borderline/Abnormal | 121 (28.8) |
| **Pro-social Behaviors** |  |
| Normal | 392 (93.3) |
| Borderline/Abnormal | 28 (6.7) |
